# Impact of COVID-19 on Medicine Lecturers’ Mental Health and Emergency Remote Teaching Challenges

**DOI:** 10.3390/ijerph18136792

**Published:** 2021-06-24

**Authors:** Carla Miguel, Luísa Castro, José Paulo Marques dos Santos, Carla Serrão, Ivone Duarte

**Affiliations:** 1 Faculty of Medicine, University of Porto, 4200-319 Porto, Portugal; 2 Department of Community Medicine, Information and Decision in Health (MEDCIS), Faculty of Medicine, University of Porto, 4200-319 Porto, Portugal; luisa.castro@fc.up.pt (L.C.); iduarte@med.up.pt (I.D.); 3 Centre for Health Technology and Services Research (CINTESIS), Faculty of Medicine, University of Porto, 4200-319 Porto, Portugal; 4 School of Health, Polytechnic of Porto, 4400-330 Vila Nova de Gaia, Portugal; 5 Department of Biomedicine, Faculty of Medicine, University of Porto, 4200-319 Porto, Portugal; jmarquessantos@med.up.pt; 6 University Institute of Maia, 4475-690 Maia, Portugal; 7 Centre for Research and Innovation in Education (inED), Porto Polytechnic School of Education, 4200-465 Porto, Portugal; carlaserrao@ese.ipp.pt

**Keywords:** COVID-19, teaching, burnout, lecturers, emergency remote teaching

## Abstract

COVID-19 has presented a novel pedagogical challenge in dealing with the sudden shift from classic instruction to emergency remote teaching (ERT). It had an impact on the well-being and mental health of lecturers, increasing burnout risk. A cross-sectional, quantitative, qualitative and analytical online study was conducted to collect participants’ sociodemographic data, responses to ERT open-ended questions and mental health assessments using relevant instruments (CBI for burnout, Resilience Scale, DASS for depression, anxiety and stress, SWLS for satisfaction with life). High personal burnout levels were found in 41.2% of participants, high work-related burnout in 37.3% and high student-related burnout in 15.7%. Satisfaction with life, sleep routine changes and stress were determinants for personal burnout; stress and resilience for work-related burnout; satisfaction of life and sleep routine changes for students-related burnout. Opportunities for pedagogical innovation were pointed out as the main advantages to ERT, while the main negative impacts were on practical lessons and social interaction. Students and lecturers’ safety and adequate institutional support might be insured, considering their expectations and needs, promoting mental health. Combining the advantages of online and traditional methods in a so-called “blended learning” approach, with close collaboration and communication between all those involved, appears to achieve better results.

## 1. Introduction

One week after the World Health Organization (WHO) declared a global pandemic due to COVID-19 in early March 2020, the Portuguese government declared a state of emergency and began limiting the rights and freedoms of its citizens, profoundly altering daily life. The proportion of people working from home rose sharply, presenting an unexpected challenge for those suddenly forced into remote work [1,2].

Most countries around the world immediately closed all educational institutions, from kindergartens to universities, and halted face-to-face education. In Portugal, all activities involving in-person teaching were suspended on 16 March, a resolution that impacted the entire academic community [3]. Educational institutions were closed without time to draw up a planned shift from classic face-to-face teaching to online-based learning in an uncertain situation. Teachers were asked to quickly implement new teaching practices to promote student learning and, at the same time, maximize student safety [4]. Higher education was forced to make abrupt changes in a few days, fully transitioning curricula from face-to-face to distance education [3,5].

The novel pedagogical challenge presented by COVID-19 gave rise to the development of emergency remote teaching (ERT), a temporary shift from classic, face-to-face teaching to an alternative, online learning approach under high-pressure circumstances. Four phases were identified in the educational response to COVID-19: (1) rapid transition to remote teaching and learning; (2) (re-) adding basics; (3) extended transition during continued turmoil; and (4) an emerging new normal [6]. The main goal of ERT is to provide temporary and reliable educational support that is easy to configure and ready to implement instead of a robust, long-term educational program. In such a narrow time frame, the transition process without ERT may become stressful and not take full advantage of the online format [7].

The process of planning, preparing, and developing a fully online higher education course is estimated to take between six to nine months and require around three iterations to become effective [7]. The minimal resources and urgency associated with quick approaches to online learning decrease its quality, as the effort required to develop a high-quality educational program cannot be met [7,8]. Barriers to the success of online teaching include a lack of technical skills, institutional support, time, cost, motivation and poor interactive relationships built between teachers and students, as well as resistance to change [9,10]. Additionally, a successful e-learning experience is the result of three factors: interaction in a socially collaborative environment; cognitive reflection and communication; and the teacher’s role in defining meaningful learning outcomes (pedagogical approach) [4]. Other important factors to take into account regarding e-learning include collaboration between learners and facilitators, taking students’ motivations and expectations into consideration, using user-friendly technology and placing the pedagogical focus on students [11]. It may also be worthwhile to combine the advantages of the two approaches in so-called “blended learning” [9].

Lecturers require the ability to multitask to manage their professional careers, additional administrative work, and their personal and social lives. Parallel to teaching, they must possess a wide range of skills and constantly keep themselves up to date and, adding research on top of this can increase stress and anxiety. When these stressors are persistent and not coupled with effective compensation mechanisms, they can lead to burnout [12,13,14].

Burnout is the result of an individual, continuous, chronic, and gradual process and is characterized by three dimensions: the feeling of energy depletion or exhaustion; a lack of interest and motivation at work; and reduced professional efficacy. It is an occupational syndrome included in the WHO’s International Classification of Diseases (ICD-11) since May 2019 [15,16].

Burnout research has been of particular interest in the field of academia. Teaching is considered to be a high-risk job, and the severity of burnout can be even higher in these professionals compared to health professionals [13]. Anxiety, irritation and sadness can be found in a burned-out teacher, which may result in somatic symptoms such as sleep disorders, headaches, gastrointestinal problems, alcohol, and drug abuse. Rather than an identifiable cause, there is a set of combined factors that makes burnout a complex and multidimensional phenomenon [13,14,16].

There has been growing awareness of the adverse influence that the environment of higher education institutions has on the mental health of academics, who have shown high levels of stress and burnout and low levels of well-being [17]. A previous COVID-19 study of 520 higher education lecturers in Portugal concluded that stress levels decrease after 60 years of age, but are higher in female lecturers and those with less than 10 and more than 30 years of professional experience [12]. Another study of teachers in Finland found that the absence of quality rest and leisure activities combined with non-restorative sleep increased the risk of burnout [18]. Regarding changes in sleep routine and quality during the COVID-19 lockdown, it was found that night-time sleep variations, poor sleep quality, a shift in sleep cycle to the delayed phase and sleep-deprivation can be associated with psychological distress in a sizable number of people [19].

A sample of 1316 lecturers from Spanish public universities was used to develop a model establishing a causal relationship between stress, burnout, emotional intelligence, and non-verbal communication. Physical activity was shown to reduce cortisol and norepinephrine levels, the two hormones produced in situations of stress and anxiety, and increase endorphins, the “happiness hormones”. Prolonged stress was confirmed to be a strong predictor of burnout syndrome [14].

Stress at work can cause a negative balance between investment and productivity, which leads to exhaustion, one of the three dimensions of burnout. According to the International Labour Organization (ILO), one in ten workers suffer from chronic stress, anxiety, burnout and depression [14,20]. In addition to its impact at the professional level, stress also affects health, personal, social and economic life [14]. Approximately 20 to 30% of teachers and an estimated 25% of lecturers report that teaching is very or extremely stressful [14,18].

However, not all individuals exposed to these challenges develop burnout or stress. Positive psychological changes and post-traumatic growth, associated with psychological resilience, have also been reported when the COVID-19 pandemic was compared to Hurricane Katrina [21]. Psychological resilience [22,23]—understood as the ability to positively adapt to situations that can potentially cause stress and anxiety, such as COVID-19—and satisfaction with life can present themselves as protective variables.

A study conducted among Dutch teachers found a relationship between the validation of personal work with higher determination, flexibility and better results [24]. Meanwhile, an online survey of 1278 Canadian teachers seeking to understand the association between burnout and resilience during the COVID-19 pandemic found that family support, exercise, healthy eating and emotional self-care (such as meditation and mindfulness) were the most protective variables [25]. Focusing on well-being and resilience under these circumstances was also found to be a key to success in a study of South African lecturers [26]. In Portugal, a study analysing variables related to the professional well-being of Portuguese teachers during the COVID-19 pandemic found that ongoing monitoring of teachers’ well-being throughout their careers is essential to help them cope with the pandemic [27]. The mediating role of subjective well-being in job burnout is well known, as is the association of poor well-being with high levels of burnout [28].

Studies on the impact of ERT on higher education are generally scarce [29], and the paucity of evidence concerning similar situations in the past makes it challenging to anticipate the future impact of these changes on the academic community [30]. Finally, the existing literature in this field is minimal.

This study aims to understand the impact of COVID-19 on lecturers from the Faculty of Medicine of the University of Porto (FMUP), a medical university in the northern region of Portugal. The goals of this study are to identify the factors that influence their susceptibility to the three dimensions of burnout and to explore lecturers’ perceptions of ERT during the pandemic crisis. It is hoped that these results might contribute towards improving the theoretical framework applied in the subsequent waves of this pandemic and helping this professional group.

## 2. Materials and Methods

### 2.1. Study Design, Context and Participants

A cross-sectional, quantitative, analytical, and qualitative study was conducted and applied to all FMUP lecturers. The study protocol follows STROBE guidelines and was approved by the Health Ethics Committee (CES) of the São João Hospital Centre/FMUP (Ref CE 98-2020 on 29 May 2020). It also follows the ethical principles enshrined in the Declaration of Helsinki (2013) and the Convention for the Protection of Human Rights and the Dignity of the Human Being in Biology and Medicine (2001).

An online questionnaire was created using Google Forms and disseminated on FMUP and the Centre for Research in Health Technologies and Services (CINTESIS)’s websites. Disclosure e-mails were also sent to all FMUP lecturers.

A pre-test was conducted on a convenience sample (*n* = 13) for comprehensiveness, to check interpretation and any format issues of the online survey.

The survey made use of a convenience sample and was available between 19 June and 31 July 2020, encompassing exams season and the first weeks of student summer holidays. This period included a declaration of national calamity and an easing of lockdown measures that followed a state of national emergency, between 18 March and 2 May. The Portuguese government gradually reduced the lockdown caused by COVID-19 [31]. These lifting of lockdown measures gave new hope to all those fearing the future and living in uncertain circumstances since March, which may have had a psychological impact. However, educational establishments remained closed, mainly in telework. FMUP was not an exception, practical lessons remained cancelled and only a few final exams were presential.

Participants were duly informed about the aims of the study, the anonymization of responses, the confidentiality of data and the mean duration time to complete the questionnaire, as well as the ability to give up at any time, and provided their free and informed consent. Fifty-one participants completed the questionnaire, and no missing data was found.

### 2.2. Variables, Measurement Instruments and Questionnaire Sections

The applied questionnaire included sociodemographic questions regarding gender, age, marital status, number, and age of children. It also included questions regarding professional experience and educational qualifications, previous teaching experience in professional virtual learning, their professional situation during the pandemic and their current mode of work. Participants were asked about their chronic diseases, mental health medications and changes to their sleep routine (number of hours, bedtime and/or wake up time). This section was followed by five open-ended questions focusing on the advantages, disadvantages, worries, challenges, and solutions of ERT. Lecturers were also asked if they agreed with the closure of higher education facilities.

In addition to the sociodemographic section, the questionnaire contained the following instruments, with authorization from the original authors: Copenhagen Burnout Inventory (CBI) [32,33]; Resilience Scale [34,35]; Depression, Anxiety and Stress Scales (DASS) [36,37]; Satisfaction with Life Scale (SWLS) [38,39].

The CBI [32], adapted and validated for the Portuguese population [33], consists of 19 items distributed across three subscales: personal burnout, composed of six items, assesses the experienced degree of physical, psychological and mental exhaustion; work-related burnout, consisting of seven items, analyses the perceived degree of physical and psychological fatigue and exhaustion while at work; and student-related burnout, consisting of six items, assesses the perceived degree of physical and psychological fatigue and exhaustion resulting from working with students. The three subscales were not presented sequentially to avoid patterns of stereotyped responses. All the items were scored on a 5-point Likert scale. The score obtained for each subscale was the average of all item scores within the subscale ranging from 0 to 100, and it was considered high-level burnout when ≥50 points [32,33]. The Cronbach’s Alpha, α, for the Portuguese version was 0.86 [33]. In this study, 0.935 was obtained for personal burnout, 0.878 for work-related burnout, and 0.830 for student-related burnout.

The Resilience Scale [34], translated and adapted for the Portuguese population [35], is composed of 25 items scored on a 7-point Likert scale, from “disagree” (1 point) to “strongly agree” (7 points). The theoretical score ranges from 25 (low resilience) to 175 (high resilience). For the Portuguese version, α was 0.89 [35] and the value obtained in this study was 0.941.

DASS [36] validated for the Portuguese population [37] consists of 21 items and is organized into three self-reported subscales to evaluate the negative emotional states of depression, anxiety and stress. Each subscale has 7 items on a 4-point Likert scale, from “did not apply to me at all” (0 points) to “applied to me very much or most of the time” (3 points). The recommended cut-offs for the conventional severity labels were used in each subscale. For the depression subscale, normal is from 0 to 4, mild is from 5 to 6, moderate from 7 to 10, severe from 11 to 13 and extremely severe from 14 to 21. For the anxiety subscale, normal is 0 to 3, mild is 4, moderate is from 5 to 7, severe is 8 and 9 and extremely severe from 10 to 21. For the stress subscale, normal is from 0 to 7, mild is 8 and 9, moderate is from 10 to 12, severe is from 13 to 16 and extremely severe from 17 to 21 [40]. In this study, α was 0.896 for the stress subscale, 0.899 for the anxiety subscale and 0.917 for the depression subscale.

The SWLS [38], validated for the Portuguese population [39], aims to assess the cognitive component of subjective well-being. It consists of 5 items on a 5-point Likert scale. In the Portuguese version, this instrument ranges between 5 to 25 points, where a higher result indicates greater satisfaction with life. The Cronbach’s Alpha for the Portuguese version was 0.77 [39] and 0.911 was obtained in this study.

### 2.3. Data Analysis and Statistical Methods

Data was exported from Google Forms in a Microsoft Excel file and analysed using SPSS^®^ Statistics (version 26.0; SPSS Inc., Chicago, IL, USA) and Jamovi software (The Jamovi project (2021). Jamovi (Version 1.6) [Computer Software], Sidney, Australia). Categorical variables were described using absolute and relative frequencies. Quantitative variables for which normality was not rejected were described by the mean and respective standard deviation. Ordinal or continuous variables not normally distributed were described by the median and the inter-quartile interval, [1stQ; 3rdQ]. The variables’ normality was assessed by analysing the histograms and confirmed using the Kolmogorov-Smirnov test.

For each outcome—personal burnout, work-related burnout, and student-related burnout—a separate multiple linear regression analysis was performed. Simple linear regressions were conducted for each independent variable to choose the relevant ones or potential predictors of burnout levels. Only the variables correlated with the outcome at *p* < 0.20 in the simple linear regression were included in each multiple linear regression analysis. Only the significant variables (*p* < 0.05) were maintained in the final multivariate models for personal, work-related, and student-related burnout. Unstandardized coefficients (β), 95% confidence intervals (95% CIs), and *p*-values were used to present the results of linear regressions. Models were evaluated using the F-statistic of the overall model test, *p*-values, and coefficients of determination (R^2^). The assumptions of the linear regression models were verified using the following three conditions: (1) histograms were used to assess the normality of residuals; (2) *T*-tests were performed to verify zero mean of the residuals; and (3) plots of residuals versus the fitted predictive values were used to check homoscedasticity. The internal consistency of each scale of the questionnaire in the study sample was assessed using Cronbach’s Alpha, α, and a value above 0.7 was considered acceptable [41]. In all tests performed, *p*-values were considered significant if they were less than 0.05.

### 2.4. Analysis of Open-Ended Questions

The answers to the open-ended questions were analysed using the six-phase Thematic Analysis method [42] supported in NVivo 12^®^ (QSR International, Burlington, MA, USA). Phases 2 through 5 were implemented by two data analysts (authors) working separately in series.

After perusing the data imported into NVivo, one data analyst generated the initial codes, which grounded code collating into subthemes and themes by the other data analyst, who also reviewed the themes along with the original subjects’ responses. Then, the data analyst named both subthemes and themes. These roles were reversed across the five open-ended questions: advantages, disadvantages, worries, challenges, and solutions regarding ERT. Finally, both data analysts met to clear up pending divergences, having agreed upon the hierarchical organization of themes, subthemes, and grounding codes. Memos describing the noteworthy aspects that emerged from the data were written for each theme and then for each open-ended question as a whole.

## 3. Results

### 3.1. Participant Characteristics

A sample of 51 participants completed the questionnaire: 35 women (68.6%) and 16 men (31.4%), with an average age of 48 ± 1 (SD) and ranging from 25 to 68 years old, all residents in the northern region of Portugal. Thirty-seven (72.5%) were married or in a civil union, 8 (15.7%) were divorced and 6 (11.8%) were single. Regarding academic qualifications, 34 (66.7%) held a doctoral degree, nine (17.6%) held a bachelor’s degree, seven (13.7%) held a master’s degree and on (2.0%) held a post-doctorate or aggregate appointment.

A set of 19 participants (37.3%) lived with people at risk for COVID-19, six (11.8%) had lost relatives or friends during the pandemic and seven (13.7%) were caregivers—four (7.8%) of whom dealt with older people and three (5.9%) with dependent people. One participant (2.0%) had asked for medical support (Family Medicine). Regarding the COVID-19 test, 15 (29.4%) had already taken one, eight (15.7%) were interested in taking one, and 28 (54.9%) were not interested in taking one. The distribution of these and other significant sociodemographic variables is shown in Table 1.

### 3.2. Levels of Burnout Dimensions and Psychological Variables

Table 2 shows the categorical results obtained in the questionnaire for personal burnout, work-related burnout, student-related burnout, resilience, stress, anxiety, and depression. Higher levels of personal burnout were found in 21 (41.2%) participants, 19 (37.3%) for work-related burnout and eight (15.7%) for student-related burnout. Resilience was moderate in 25 (49.0%) and high in 19 (37.3%) participants. Anxiety (84.3%), depression (82.4%) and stress (78.4%) were normal in most participants, as shown in Table 2. The lecturers from our sample showed a median [1stQ; 3rdQ] of 20 (17; 23) points on the scale of satisfaction with life.

### 3.3. Factors Associated with Different Burnout Subscales: Simple and Multiple Linear Regression Analyses

For each sociodemographic, professional, and psychological variable considered relevant or a potential predictor of burnout, a simple linear regression analysis was computed with results presented in Table 3. For each burnout dimension, significant variables from the simple regressions at a level of *p* < 0.2 (highlighted in bold in Table 3) were selected for the multiple regression model.

Professional experience, satisfaction with life, the current mode of professional activity, sleep routine changes, anxiety, depression, stress, and resilience were included in the multiple regression model for personal burnout.

Professional experience, satisfaction with life, the current mode of professional activity, sleep hours, sleep routine changes, anxiety, depression, stress and resilience were included in the multiple model for work-related burnout.

Satisfaction with life, the current mode of professional activity, chronic diseases, sleep routine changes, anxiety, depression, stress, and resilience were included in the multiple regression model for student-related burnout.

For personal burnout, three variables were significant in the final multivariate linear regression model: satisfaction with life, sleep routine changes and stress explaining approximately 56% of the total data variance in this burnout dimension (Table 4). For work-related burnout, stress and resilience were significant in the final multivariate linear regression model, explaining approximately 55% of the total data variance (Table 4). Regarding student-related burnout, satisfaction with life and sleep routine changes were found to be significant in the final multivariate linear regression model, explaining approximately 31% of the total data variance (Table 4).

Higher levels of satisfaction with life were significantly associated with lower levels of two burnout dimensions: β = −1.46 (*p* = 0.041) for personal burnout and β = −2.38 (*p* < 0.001) for student-related burnout. Sleep routine changes were significantly associated with higher levels of burnout, and those participants scored, on average, 12.69 points higher in personal burnout levels and 10.81 points higher in student-related burnout levels, compared to lecturers without changes in their sleep routine. Higher levels of stress were also associated with higher levels of personal burnout, β = 2.66 (*p* < 0.001), and work-related burnout, β = 2.18 (*p* < 0.001). In addition, higher levels of resilience were associated with lower levels of work-related burnout: participants scored, on average, 0.39 points less in this burnout dimension, for each point increase in the resilience score (Table 4).

Given the sample size, the achieved power in the multiple regression was computed using G*Power online software [43]. In the final multiple regression using 2 or 3 predictors, a sample of 51 participants, a significance level of 0.05 and an effect size of 0.25 (considered between medium and large [44]), the powers obtained were 0.88 or 0.83, respectively.

### 3.4. ERT from the Lecturers’ Perspective: A Qualitative Analysis

The questionnaire included five open-ended questions focusing on the advantages, disadvantages, worries, challenges, and solutions of ERT. The main themes that emerged from the answers for each topic are summarized in Table 5 and Table 6.

In terms of advantages, the most addressed theme is the class itself, followed by the lecturer, the student, and the impact on society. Some respondents claim that the only benefit of ERT is that it allows classes to continue during the lockdown. If the class itself was already the epicentre of classroom lecturing, it continues to deserve the spotlight among the advantages of ERT. The most referred to advantages include flexibility (specifically, time flexibility) and the opportunity for pedagogical innovation, such as making recordings of the classes available asynchronously, providing more diverse study materials, or more easily hosting guest speakers. There is the perception that all these aspects contribute to more effective classes, which translates into better results. Time management, convenience, and comfort due to the elimination of the daily commute and working from home are also positives mentioned by respondents. They also highlighted greater student autonomy and responsibility and the ability to stay home (for those from different cities). Finally, participants recognize that ERT has contributed to controlling the pandemic, which is important for society.

As for the disadvantages, classes and socialisation were the primary concern, but the lecturer theme was also addressed. Class dynamics are an issue in ERT, as it is difficult to motivate students to participate when they often have their webcams off. The inability to hold practical classes, considered to be of major importance in medical education, was recurrently identified as a disadvantage, and one to which ERT does not yet have suitable solutions. All these issues compromise the teaching-learning process. The lack of socialisation was another repeated drawback, mainly concerning the interaction between lecturers and students. As for the lecturer theme, reported difficulties were related with the adaptation to a new system and technologies without proper institutional support.

The dominant theme that emerged during data analysis of the worries surrounding ERT was the teaching-learning process, followed by socialisation and the students. The teaching-learning process worries centre on two issues: poor practical classes, which lack an actual practical component; and, similarly, poor contact with patients and clinical cases, both precluding the necessary acquisition of knowledge and skills required to become a doctor. Student assessment is also a worry, mainly due to the possibility of cheating. Student demotivation was another concern, as was poor socialisation in ERT environments. The dehumanization and depersonalization of teaching was also a worry for the lecturers surveyed, and they feared an increased risk of mental illness associated with this situation.

The challenges were closely connected with the worries that emerged. The main one was how to re-invent practical classes, mostly in laboratories, and contact with patients and clinical cases, primarily in the hospital. Other identified challenges were how to ensure effective learning and student motivation, as well as monitoring of mental health to avoid overloading students and lecturers with personal and work issues, protecting their psychological well-being and preventing burnout.

Study participants were also invited to contribute with solutions. Their answers were classified into two themes: the teaching-learning process and the lecturer. Remarkably, there are respondents whose contribution was to provide no solutions; since lecturers and students will return to the traditional classes as soon as the pandemic is eradicated, they argued, it would be a waste of time. The proposed solution to the teaching-learning process should focus on a combined method that still encompasses practical classes and live clinical cases. Considering the answers, this method is known as “blended learning” (b-learning) and combines online classes (synchronous and asynchronous) with in-situ ones. Practical education, conducting experiments, contact with patients, etc., would be taught in in-situ classes. The theoretical content could be delivered through online classes, which could be asynchronous (increased weight) and/or synchronous (less weight). Still in this vein, respondents refer to using active classroom pedagogies, materials that benefit from digital formats (like videos) and keeping webcams on. But the solutions must also address lecturers’ involvement, as they must invest more time in designing the classes and study materials, providing extra support to students through individual or small group tutorials, for example, and learning about the world of digital technologies for education.

Regarding the closure of higher education facilities, 10 participants (19.6%) agreed with the decision, 17 (33.3%) neither agreed nor disagreed and 24 (47.1%) disagreed. The public health preoccupation in reducing the risk of the COVID-19 infection was the most consensual justification for the decision. Lecturers who did not agree with this decision argued that the situation must be faced, due to the temporal unpredictability of eradication of the virus, and it was better to return to face-to-face education with mitigation measures that guarantee the safety of those involved, or to at least try a mixed regime with face-to-face practical classes and remote theoretical lessons. The participants considered remote education to be non-viable in the long run and something that should only be used as a temporary and complementary resource, since traditional education provides a unique experience. Lecturers who remained neutral considered the evolution of the pandemic to be the main conditioning factor for the type of teaching regime to be adopted, in addition to the logistical conditions and characteristics of each course and curricular unit, always maintaining the safety conditions of all those involved.

## 4. Discussion

The COVID-19 pandemic has had a relevant impact on the well-being and mental health of lecturers around the world, including by increasing the risk of burnout [14]. The immediate shift from the classic, face-to-face teaching approach to ERT seems to have contributed to this complex and multidimensional phenomenon. A cross-sectional, quantitative, analytical, and qualitative study was conducted using an online questionnaire that included sociodemographic questions, five open-ended questions focused on ERT and mental health assessment instruments (CBI for burnout, the Resilience Scale, DASS for depression, anxiety and stress, and SWLS for satisfaction with life).

Our findings show high personal burnout levels in 41.2% of participants, high work-related burnout in 37.3% and high student-related burnout in 15.7%. These results were average, putting them into alignment with previous studies, such as one with 300 university lecturers, readers and professors in India [45] and another with 648 university academicians in Turkey [46]. The questionnaire was applied during exams season and after the initial societal adaptation to the COVID-19 pandemic. The fact that participants had already had to adjust their parental, family and professional responsibilities to the new reality months ago may explain the average burnout levels found.

The results of the psychological assessment instruments show moderate resilience in 49.0% of the sample, high resilience in 37.3% and normal levels for anxiety (84.3%), depression (82.4%) and stress (78.4%) in most of the participants. These levels are lower than those found in other studies, such as one with 200 Libyan schoolteachers that found 44.5% for depression, 56% for anxiety and 39.5% for stress [47]. Another study with 2530 students and staff at a Spanish university found 35.18%, 48.10% and 40.32% for anxiety, depression and stress scores, respectively [48]. These results also support the need for close collaboration and communication between all those involved in teaching and learning, as they are all affected by these variables and can make a relevant contribution towards a more effective system. In addition, around 65% of participants had over 15 years of teaching experience and, as already mentioned, the initial adaptation to COVID-19 had already taken place, which may explain the enhanced ability to cope with anxiety and stress.

The effect of sociodemographic and psychological variables on the three dimensions of burnout were explored, and some of these variables were significant in the final multiple linear regression models. Satisfaction with life, sleep routine changes and stress accounted for more than half of the personal burnout variance, approximately 56%. Stress and resilience accounted for approximately 55% of the total data variance of this work-related burnout. Satisfaction with life and sleep routine were significant for student-related burnout, explaining approximately 31% of total data variance of this dimension.

Evidence shows that sleep routine changes lead to fatigue, tiredness and exhaustion and can increase the risk of burnout [14,18,19]. In our study, this variable was present in two of the three models, where lecturers with sleep routine changes scored, on average, 12.69 points higher in personal burnout levels and 10.81 points higher in student-related burnout than those without changes. However, there is a lack of quantitative evidence describing the relationship between burnout and sleep routine changes and further research could be done to better characterize this association.

Stress and burnout are different, but closely associated with identical work-based psychosocial factors [49], and our results are aligned with this association. Stress, as a tendency to overreact to a stressful event, was presented in the final model of personal burnout and work-related burnout. We cannot forget that approximately 25% of lecturers report that teaching is very or extremely stressful [18], reinforcing this idea.

Satisfaction with life and subjective well-being are also known to play a mediating role in job burnout by reducing its levels [27,28], which our results support with this variable present in two models: personal burnout and student-related burnout. Also, resilience, as the ability to positively adapt to situations that can potentially cause stress and anxiety, appeared to be associated with work-related burnout [25,26]. Health programs that promote satisfaction with life and train resilience in lecturers should be considered by educational establishments, as psychological resilience and satisfaction with life seem to be protective variables against burnout.

This study also sought to understand lecturers’ opinions and suggestions regarding ERT. Considering the advantages, the most mentioned theme was the class itself having more flexibility and opportunity for pedagogical innovation, followed by comfort and convenience for the lecturer, student autonomy and responsibility (which is aligned with the literature [7,8,11]) and the societal impact of reducing the risk of COVID-19 in the current pandemic context. Regarding the disadvantages, the inability to hold practical classes—considered to be of major importance in medical education—and socialisation came first, but the lecturer theme was also addressed in terms of the extra work required to adapt to the new teaching system. The most common worry theme that emerged during the data analysis was the teaching-learning process, followed by socialisation and students. The teaching-learning process worries concentrate around the poor practical classes and poor contact with patients and clinical cases. All of these barriers and worries have already been mentioned in previous research [9,10,11].

Online learning is not an effective teaching method for every student in every learning context, and a combined method—practical lessons would be taught in-situ while theoretical content would be delivered through online classes—appears a hopeful solution, which is in alignment with previous research [9]. It is also relevant to emphasize lecturers’ additional availability to monitor their students more closely. Therefore, the results were aligned with the three-factor model for a successful higher education e-learning experience: interaction in a socially collaborative environment, cognitive reflection and communication and the lecturer’s role in defining meaningful learning outcomes [4].

Almost half of the participants disapproved of the decision to close higher education facilities, which are an important part of the process to improve teaching strategies and help lecturers deal with these pressing circumstances. Lecturers pointed out that training and institutional support might play a key role in improving Information Technology (IT) skills, as is already known [9,26]. Online platforms, material support for distance learning and even a possible reformulation of the study plan could have an important effect on lecturers’ subjective well-being and, consequently, improve the learning process, as has been reported in previous research [4,11] and explored in previous paragraphs.

Although the results obtained were able to provide useful information, some limitations can be pointed out. This study was shared online and applied to a convenience sample, with potential snowball sampling, but it could have limited the questionnaire’s accessibility and not have reached most of the study population. Lecturers might not regularly visit the official websites used to share the study. Disclosure emails were sent out trying to overcome this difficulty, but lecturers might not read this kind of e-mails. Furthermore, the questionnaire was shared during exams season, a period associated with accumulated tiredness and that could have led to a smaller than expected number of answers. It is important to remember that most of these lecturers are healthcare workers, and during this time they had an increased workload, and other questionnaires to answer because many studies were also being developed. Another limitation is the observational nature of this cross-sectional study, which does not establish causal relationships between variables, but rather provides suggestions of causality through the associations found that can be further explored in future studies and experiments. The sample size was small and may explain the lack of more variables to justify burnout and limits the external validity of our study.

Further research could benefit from the topics addressed in the open-ended questions regarding ERT, improving their results with important items that were forgotten or not referred to despite their importance for lecturers. These could provide a good starting point for additional quantitative studies to better characterize and understand how the teaching-learning process could be improved. More additional research could be done to better explain the association between burnout and the variables that the multiple linear regression models identified, allowing comparative results even at the end of the COVID-19 pandemic.

## 5. Conclusions

High levels of personal burnout were found in 41.2% of lecturers, which must be recognized and 16% of participant lecturers experienced high levels of student-related burnout. These teachers will tend to be less available to the student for pedagogical involvement, compromising teaching and learning. Satisfaction with life, sleep routine changes and stress were associated with personal burnout, accounting for approximately 56% of its variance in the final multiple linear regression model. Stress and resilience were associated with work-related burnout, accounting for approximately 55% of its variance in the final multiple linear regression model obtained. Satisfaction with life and sleep routine changes were associated with student-related burnout, accounting for approximately 31% of its variance in the final multiple linear regression model. Satisfaction with life and resilience appeared to be a protective variable, whilst stress and sleep routine changes were negatively associated with burnout.

Safety of all involved might be insured, considering the expectations and needs of students and lecturers, with adequate institutional support, and promoting mental health. Healthy routines, leisure activities and sports are essential and should be part of everyday life, combined with personal, social, and institutional support. As resources could be particularly scarce during a serious pandemic situation, like this one caused by COVID-19, timely psychological support should be taken into account to promote mental health [50] at home or work and improve access to mental health services. Telemedicine such as psychiatric teleconsultation, videoconferencing and informal support groups should be considered as potential therapy options. Coping and self-care mechanisms seem to be considered and should be encouraged. Institutional politics may play a relevant role to prevent burnout by providing solutions for the organizational, social, and emotional needs of lecturers, combined with strategies to control or avoid work overload. It is crucial to implement mitigation strategies to promote lecturers’ health and, consequently, guaranteeing teaching quality. Thus, institutions should provide resilience and self-care development programs and allow lecturers daily time for pleasurable activities.

Opportunities for pedagogical innovation were pointed out as the main advantages of ERT, as well as the societal impact of reducing the risk of COVID-19 during the current pandemic context. When considering the disadvantages, practical lessons and social interaction are the primary concern. Aligned with this, finding a perfect solution or decision may be a complex task, but close collaboration and communication between all those involved in teaching appears to be a significant option. This extraordinary situation is an opportunity to increase teaching flexibility, and the opportunity to identify the best strategies and plan the most effective online-based learning environments should be seized. When the COVID-19 pandemic is over, the practical knowledge acquired from this situation might be used to improve teaching methodologies instead of simply returning to the traditional teaching-learning process. Remote education, although far from perfect when used on its own, could be used as a complementary resource to expand the potential of both online and in-situ learning.

## Figures and Tables

**Table 1:** Distribution of sociodemographic variables.

Variables	*n* (%)
**Gender**	
Women	35 (68.6)
Men	16 (31.4)
**Marital Status**	
Single	6 (11.8)
Married/Civil Union	37 (72.5)
Divorced/Separated	8 (15.7)
**Children**	
No	11 (21.6)
≤12 years old	19 (37.3)
>12 years old	21 (41.2)
**Academic Qualifications**	
Bachelor’s degree	9 (17.6)
Master’s degree	7 (13.7)
Doctoral degree	34 (66.7)
Post-doctoral or aggregation	1 (2.0)
**Professional Experience**	
≤5 years	7 (13.7)
6 to 15 years	11 (21.6)
>15 years	33 (64.7)
**Previous Experience with Virtual Learning**	
Yes	12 (23.5)
No	39 (76.5)
**Professional Activity During State of Emergency**	
Active at workplace	19 (37.3)
Active at telework	28 (54.9)
Layoff	2 (3.9)
Maternity/paternity license	1 (2.0)
Other	1 (2.0)
**Current Mode of Professional Activity**	
Only at workplace and partial telework	32 (72.5)
Only at telework	14 (27.5)
**Chronic Diseases**	
Yes	11 (21.6)
No	40 (78.4)
**Household**	
Living alone	6 (11.8)
Not living alone	45 (88.2)
**Living with People at Risk of COVID-19**	
Yes	19 (37.3)
No	32 (62.7)
**Death of a Relative or Friend during the Pandemic**	
Yes	6 (11.8)
No	45 (88.2)
**Asked for Medical Support**	
Yes (Family Medicine)	1 (2.0)
No	50 (98.0)
**Have Taken a COVID-19 test**	
Yes	15 (29.4)
No, and no interest in taking one	28 (54.9)
No, but interested in taking one	8 (15.7)
**Sleep Hours**	
<6 h	6 (11.8)
6 to 8 h	43 (84.3)
>8 h	2 (3.9)
**Sleep Routine Changes**	
Yes (number of hours, bedtime and/or wake up time)	26 (51.0)
No	25 (49.0)
**Caregiver during Pandemic**	
Of older people	4 (7.8)
Of dependent people	3 (5.9)
Total	7 (13.7)

**Table 2:** Burnout, resilience, stress, anxiety, and depression levels.

Personal Burnout (CBI)	*n* (%)
High levels	21 (41.2)
Not high levels	30 (58.8)
**Work-Related Burnout (CBI)**	
High levels	19 (37.3)
Not high levels	32 (62.7)
**Student-Related Burnout (CBI)**	
High levels	8 (15.7)
Not high levels	43 (84.3)
**Resilience**	
High	19 (37.3)
Moderate	25 (49.0)
Reduced	7 (13.7)
**Stress (DASS)**	
Normal	40 (78.4)
Mild	4 (7.8)
Moderate	6 (11.8)
Severe	0 (0.0)
Extremely severe	1 (2.0)
**Anxiety (DASS)**	
Normal	43 (84.3)
Mild	4 (7.8)
Moderate	3 (5.9)
Severe	0 (0.0)
Extremely severe	1 (2.0)
**Depression (DASS)**	
Normal	42 (82.4)
Mild	4 (7.8)
Moderate	3 (5.9)
Severe	1 (2.0)
Extremely severe	1 (2.0)

**Table 3:** Regression unstandardized coefficients (*β*) for CBI dimensions as outcomes and socio-demographic, professional and emotional variables as predictors in simple linear regression analysis models.

Variable	Personal Burnout *β* [95% CI]	Work-Related Burnout *β* [95% CI]	Student-Related Burnout *β* [95% CI]
**Gender**			
Men	Reference	Reference	Reference
Women	6.35 [−7.28; 20.0] *p* = 0.354	2.03 [−9.51; 13.6] *p* = 0.726	−5.86 [−16.9; 5.14] *p* = 0.289
**Children**			
≤12 years old	Reference	Reference	Reference
No	−1.32 [−18.6; 15.97] *p* = 0.879	0.12 [−14.4; 14.61] *p* = 0.987	−7.08 [−21.0; 6.82] *p =* 0.311
>12 years old	−6.47 [−20.9; 7.97] *p* = 0.372	−5.76 [−17.9; 6.36] *p* = 0.344	−6.46 [−18.1; 5.15] *p* = 0.269
**Professional Experience**			
≤5 years	Reference	Reference	Reference
6 to 15 years	**30.8 [10.82; 50.8]** ***p* = 0.003**	**22.59 [5.24; 39.9]** ***p* = 0.012**	9.20 [−8.63; 27.0] *p* = 0.305
>15 years	10.2 [−6.98; 27.4] *p* = 0.238	8.52 [−6.41; 23.5] *p* = 0.257	5.66 [−9.68; 21.0] *p* = 0.462
**Previous Experience with Virtual Learning**			
No	Reference	Reference	Reference
Yes	−5.13 [−20.1; 9.84] *p* = 0.494	−0.847 [−13.5; 11.8] *p* = 0.893	−0.641 [−12.8; 11.5] *p* = 0.916
**Satisfaction with Life**	**−2.80 [−4.21; −1.39]** ***p* < 0.001**	**−2.56 [−3.71; −1.41]** ***p* < 0.001**	**−2.18 [−3.34; −1.02]** ***p* < 0.001**
**Current Mode of Professional Activity**			
Only at workplace and partial telework	Reference	Reference	Reference
Only at telework	**−14.2 [−27.9; −0.45]** ***p* = 0.043**	**−12.2 [−23.7; −0.71]** ***p* = 0.038**	**−11.5 [−22.6; −0.39]** ***p* = 0.043**
**Chronic Diseases**			
No	Reference	Reference	Reference
Yes	−5.64 [−21.1; 9.78] *p* = 0.466	−5.55 [−18.5; 7.39] *p* = 0.393	**−12.4 [−24.4; −0.044]** ***p* = 0.044**
**Household**			
Living alone	Reference	Reference	Reference
Not living alone	−3.43 [−23.2; 16.3] *p* = 0.729	−2.98 [−19.6; 13.6] *p* = 0.721	−8.89 [−24.7; 6.94] *p* = 0.264
**Sleep Hours**			
<6 h	Reference	**Reference**	Reference
6 to 8 h	−7.49 [−27.3; 12.3] *p* = 0.449	**−11.5 [−28.0; 4.98]** ***p* = 0.167**	−1.87 [−17.9; 14.1] *p* = 0.815
>8 h	−22.22 [−59.2; 14.8] *p* = 0.233	−16.7 [−47.6; 14.24] *p* = 0.284	−18.06 [−48.0; 11.9] *p* = 0.231
**Sleep routine changes**			
No	Reference	Reference	Reference
Yes	**14.1 [2.02; 26.2]** ***p* = 0.023**	**7.04 [−3.50; 17.6]** ***p* = 0.186**	**8.04 [−2.02; 18.1]** ***p* = 0.115**
**Anxiety**	**3.15 [1.39; 4.91]** ***p* < 0.001**	**2.82 [1.37; 4.28]** ***p* < 0.001**	**1.13 [−0.44; 2.70]** ***p* = 0.153**
**Depression**	**3.22 [1.75; 4.69]** ***p* < 0.001**	**3.19 [2.06; 4.33]** ***p* < 0.001**	**2.05 [0.77; 3.32]** ***p* = 0.002**
**Stress**	**3.66 [2.53; 4.79]** ***p* < 0.001**	**2.94 [1.96; 3.93]** ***p* < 0.001**	**1.52 [0.35; 2.69]** ***p* = 0.012**
**Resilience**	**−0.50 [−0.81; −0.19]** ***p* = 0.002**	**−0.60 [−0.83; −0.37]** ***p* < 0.001**	**−0.38 [−0.64; −0.13]** ***p* = 0.004**

**Table 4:** Regression unstandardized coefficients (*β*) for CBI subscales as outcomes and socio-demographic, professional, and emotional variables as predictors from multiple linear regression models.

Variable	Personal Burnout *β* [95% CI]	Work-Related Burnout *β* [95% CI]	Student-Related Burnout *β* [95% CI]
**Satisfaction with Life**	−1.46 [−2.85; −0.06] *p* = 0.041	-	−2.38 [−3.49; −1.27] *p* < 0.001
**Sleep Routine Changes**			
No	Reference	-	Reference
Yes	12.69 [3.54; 21.84] *p* = 0.008		10.81 [2.07; 19.55] *p* = 0.016
Stress	2.66 [1.34; 3.99] *p* < 0.001	2.18 [1.20; 3.16] *p* < 0.001	-
Resilience	-	−0.39 [−0.61; −0.17] *p* < 0.001	-
**R^2^**	0.555	0.545	0.314
**F (*p*-value)**	19.5 (*p* < 0.001)	28.7 (*p* < 0.001)	11.0 (*p* < 0.001)

**Table 5:** Advantages and disadvantages of ERT: results of qualitative analysis.

Advantages	Disadvantages
**The Class**	**The Class**
time flexibility;	difficult to motivate students to participate;
opportunity for pedagogical innovation;	webcams off;
diverse studying materials;	practical classes
invite guest lecturers	
**The Lecturer**	**The Lecturer**
time management;	extra work;
commute/travel savings;	adaptation to technology
convenience;	
comfort (home)	
**Societal impact**	**Socialisation**
contributes to controlling the pandemic	poor interactions between lecturers-students
**The student**	
improves autonomy;	
improves responsibility;	
convenience	
**Just to overcome the pandemic**	

**Table 6:** Worries, challenges and possible solutions for ERT: results of qualitative analysis.

Worries	Challenges	Solutions
**Teaching-Learning System**	**Teaching-Learning System**	**Teaching-Learning System**
poor practical classes; poor contact with patients; lack of clinical cases; student assessment	re-invent practical classes (labs); make contact with patients possible; enable real clinical cases; effective student learning	combine practical and theoretical classes; blended learning; practical classes live and in-situ; theoretical classes online (asynchronous and/or synchronous); active pedagogies; digitally-based study materials (focus on videos); webcams on
**The Lecturer**	**The Lecturer**	**The Lecturer**
risk of mental illness	keep mentally healthy	improve class design; improve study materials; close monitoring of students; add individual/group tutorial sessions; learn digital technologies for education
**The Student**	**The Student**	
demotivation; risk of mental illness	motivate students; keep mentally healthy	
**Socialization**		
lack of human contact; dehumanization; depersonalization		

## Data Availability

The exact data can be obtained from the corresponding author.
